# Use of Patient-Reported Symptoms from an Online Symptom Tracking Tool for Dementia Severity Staging: Development and Validation of a Machine Learning Approach

**DOI:** 10.2196/20840

**Published:** 2020-11-11

**Authors:** Aaqib Shehzad, Kenneth Rockwood, Justin Stanley, Taylor Dunn, Susan E Howlett

**Affiliations:** 1 DGI Clinical Inc Halifax, NS Canada; 2 Geriatric Medicine Research Unit Nova Scotia Health Authority Halifax, NS Canada; 3 Division of Geriatric Medicine Dalhousie University Halifax, NS Canada; 4 Department of Pharmacology Dalhousie University Halifax, NS Canada

**Keywords:** dementia stage, Alzheimer disease, mild cognitive impairment, machine learning

## Abstract

**Background:**

SymptomGuide Dementia (DGI Clinical Inc) is a publicly available online symptom tracking tool to support caregivers of persons living with dementia. The value of such data are enhanced when the specific dementia stage is identified.

**Objective:**

We aimed to develop a supervised machine learning algorithm to classify dementia stages based on tracked symptoms.

**Methods:**

We employed clinical data from 717 people from 3 sources: (1) a memory clinic; (2) long-term care; and (3) an open-label trial of donepezil in vascular and mixed dementia (VASPECT). Symptoms were captured with SymptomGuide Dementia. A clinician classified participants into 4 groups using either the Functional Assessment Staging Test or the Global Deterioration Scale as mild cognitive impairment, mild dementia, moderate dementia, or severe dementia. Individualized symptom profiles from the pooled data were used to train machine learning models to predict dementia severity. Models trained with 6 different machine learning algorithms were compared using nested cross-validation to identify the best performing model. Model performance was assessed using measures of balanced accuracy, precision, recall, Cohen κ, area under the receiver operating characteristic curve (AUROC), and area under the precision-recall curve (AUPRC). The best performing algorithm was used to train a model optimized for balanced accuracy.

**Results:**

The study population was mostly female (424/717, 59.1%), older adults (mean 77.3 years, SD 10.6, range 40-100) with mild to moderate dementia (332/717, 46.3%). Age, duration of symptoms, 37 unique dementia symptoms, and 10 symptom-derived variables were used to distinguish dementia stages. A model trained with a support vector machine learning algorithm using a one-versus-rest approach showed the best performance. The correct dementia stage was identified with 83% balanced accuracy (Cohen κ=0.81, AUPRC 0.91, AUROC 0.96). The best performance was seen when classifying severe dementia (AUROC 0.99).

**Conclusions:**

A supervised machine learning algorithm exhibited excellent performance in identifying dementia stages based on dementia symptoms reported in an online environment. This novel dementia staging algorithm can be used to describe dementia stage based on user-reported symptoms. This type of symptom recording offers real-world data that reflect important symptoms in people with dementia.

## Introduction

### Background

People living with dementia experience a variety of symptoms. These symptoms cross several domains beyond cognition, including executive function (eg, planning [1]), behavior (eg, agitation [2]), and physical manifestations (eg, mobility [3]). This heterogeneity of symptoms is further increased by changes in daily occurrence and manifestation. Furthermore, these combinations can vary both between people and within people across time [4-6]. This variability can be informative. Hallucinations, for example, have been reported in all stages of Alzheimer disease but most commonly at later stages [7]. In contrast, in people with Lewy body dementia, they can be a presenting feature [8]. The complex nature of dementia poses diagnostic and management challenges for health care professionals [9,10]. A key strategy is recognizing patterns, which forms the basis of dementia staging. Pattern recognition can be enhanced by tracking dementia symptoms early in the course of progressive cognitive impairment. This is especially useful when employing an approach that allows common but under-studied symptoms (eg, verbal repetition [11] or misplacing objects [12]), which may nevertheless be informative when assembled in an accessible fashion [5,6] or respond to treatment [13], to be recognized and evaluated.

### Requirement for Dementia Staging Tools

To allow individual applicability, any treatment approach must consider the person’s dementia stage [14]. Several clinician-facilitated dementia tools allow face-to-face staging, including the Global Deterioration Scale (GDS) [15], the Functional Assessment Staging Test (FAST) [16], the Dependence Scale [17], and the Clinical Dementia Rating Scale Sum of Boxes [18]. Defining dementia from unadjudicated online encounters (ie, where people living with dementia symptoms or their care partners track their symptoms in a web-based tool) is an important challenge that could improve both early detection and treatment evaluation [19]. Even so, dementia staging from solely online interactions has rarely been explored [20-22].

Online symptom tracking tools are common ways to help health care professionals understand dementia symptoms. They can also be valuable as education tools. SymptomGuide Dementia (DGI Clinical Inc) is an online dementia symptom tracking tool that provides a library of common and distressing symptoms. It serves as an educational tool and allows a user to identify symptoms of concern and track their change over time [5,23]. Earlier, we developed an algorithm to stage dementia severity into 4 levels of cognitive impairment for use with SymptomGuide Dementia or other similar databases using clinician-staged symptom profiles of 320 people [24]. Here, we aimed to develop a new staging algorithm using machine learning techniques with training data from a larger and more diverse set of clinical data and to validate this approach with well-established clinical dementia staging tools.

## Methods

### Participants and Procedure

Data for this study were obtained from a tertiary care memory clinic in Halifax, Nova Scotia, Canada from 2007 to 2013 as well as data from a study in long-term care, and an open-label trial of donepezil in vascular and mixed dementia (VASPECT) clinical trial [25,26]. Data from patients and family members (care partners) were collected using SymptomGuide Dementia in its electronic (web-based) or paper format. In addition, participants (N=717) underwent standard clinical assessments, including staging of dementia with one of two clinical tests, the GDS or the FAST. Both GDS and FAST have excellent reliability and validity [16,27]. Additionally, FAST stages have been shown to be concordant with GDS stages, and a correlation of 0.9 has been observed between them [28]. The GDS and FAST scores were interpreted as follows: a score of 3 indicated mild cognitive impairment, a score of 4 indicated mild dementia, a score of 5 indicated moderate dementia, and a score of 6 indicated severe dementia. These stages were used as target variables for classification prediction. All 4 stages were treated as discrete; therefore, discriminative models were used to perform the classification task. Only data collected at baseline (first visit) for each participant were prepared and used to train the models.

A web-based symptom tracking tool aimed to support caregivers of persons living with dementia, SymptomGuide Dementia, was used for data capture and storage for data obtained from the 3 sources. The symptoms can be either selected from an existing library of standardized symptoms or created by the caregiver. For each of the standardized symptoms, several plain-language descriptors are present. These provided another submenu for selection by the user. For each symptom selected, users were asked to indicate the frequency of the symptom and rank all the symptoms from most to least important. Users were also asked to input demographic information (eg, age and gender) and health-related information (eg, duration since first symptom), which was attached to their symptom profiles. Symptom information for each participant in the 3 sources was coded in the same format as represented in the online database. We, therefore, refer to *participants* when describing their characteristics and *user profiles* in relation to the representation of their symptoms.

### Data Preparation

Users who did not select at least one symptom from the existing library of standardized symptoms were excluded from the analysis. Any patient age reported as less than 40 years was replaced with the group average for the respective stage. This was done with the assumption that the survey question was misinterpreted, and the reported age was the care partner’s age not the age of the participant with dementia. Each symptom was represented by the ratio of descriptors selected for that symptom to the total number of descriptors selected across all symptoms by the participant. In addition to individual symptoms, the ratio of selected descriptors and ratio of reported frequency of all symptoms were grouped into the following 5 domains: Behavioral Function, Cognitive Function, Daily Function, Executive Function, and Physical Manifestations for each participant [29]. Finally, age and the duration of symptoms were also included as features (variables used for prediction). All features were continuous except for the duration of symptoms which was treated as categorical data (I don't know, 1-3 months, 3-6 months, 6-12 months, 1-2 years, 1 year or more). Of the 56 symptoms in the standardized menu in SymptomGuide Dementia, 37 symptoms were selected to be included in the final set of features. This was accomplished by pruning symptoms based on a minimum occurrence of at least 15 times. As before in the algorithm developed with 320 users [24], here we maintained the 4 common clinical classifications of mild cognitive impairment, and mild dementia, moderate dementia, and severe dementia. Of the 717 users, a majority (332, 46.3%) were clinically staged as having mild dementia with the FAST or GDS, 133 (18.5%) as having mild cognitive impairment, 138 (19.2%) as having moderate dementia, and 114 (15.8%) as having severe dementia.

Since the different dementia stages were not equally represented in the data set, the minority stages (eg, mild cognitive impairment, moderate dementia, and severe dementia) were oversampled and the most represented stage (mild dementia) was undersampled in the machine learning pipeline. Oversampling was done with the borderline variant of the synthetic minority oversampling technique algorithm with a target of increasing the minority stage sizes by approximately 1.45 times their original size [30]. Undersampling was done with the neighborhood cleaning rule algorithm that focuses on data cleaning rather than data reduction. This technique has been previously shown to improve identification of minority classes in machine learning [31].

### Building the Model

The models were adjudicated and iterated using measures of balanced accuracy, precision (also known as positive predictive value), recall (also known as sensitivity), Cohen κ, area under the receiver operating characteristics curve (AUROC), and area under the precision-recall curve (AUPRC). Balanced accuracy in this study was the average of individual accuracy for each stage [32]. In a balanced data set, this score would represent the accuracy. Data were stratified by stage and randomly split with 70% of the data used as a training data set (n=502) and 30% of the data used as a test data set (n=215) for validation.

**Figure 1:**
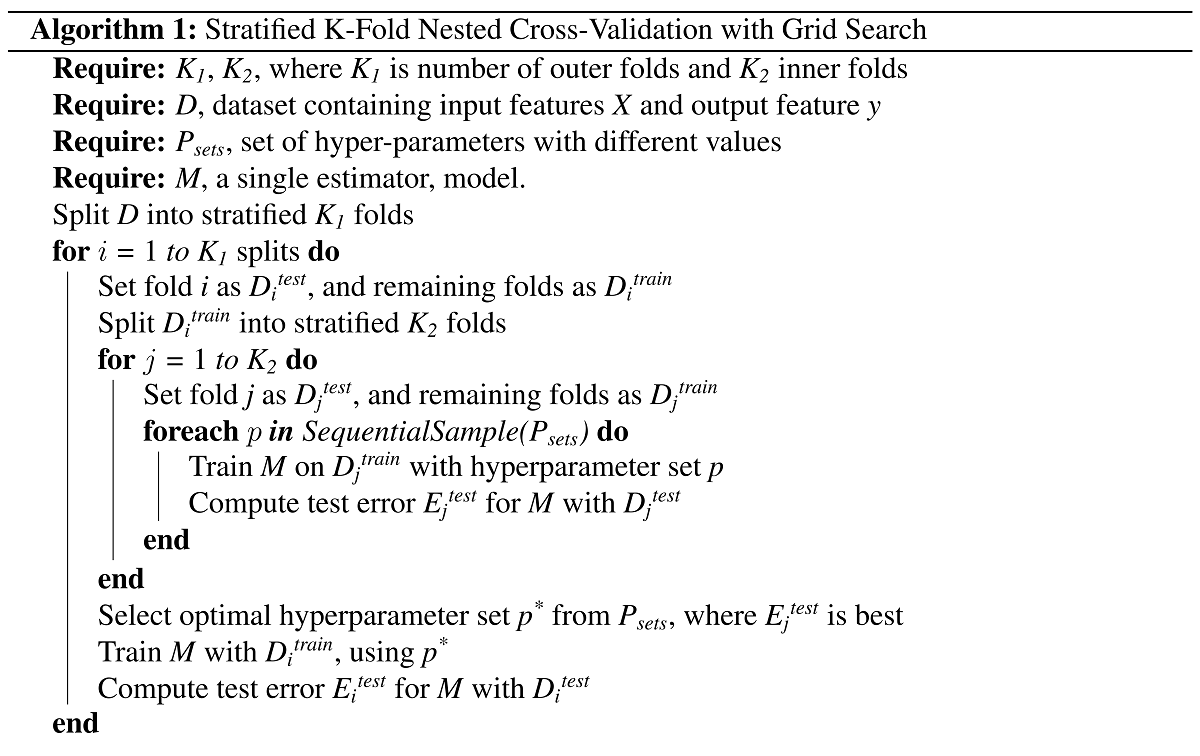
Pseudocode representation of the nested cross-validation procedure used during model selection trials.

The use of a single set of data to conduct both model selection and model training can lead to overfitting and selection bias [33]. To address this, we used a nested cross-validation approach as described in Figure 1. The average inner cross-validation estimates of the primary selection criterion were maximized by selecting optimal hyperparameters from a range of possible values. The inner and outer cross-validation loops were repeated 3 times to account for variance arising from choice of data set splits [34,35]. We used 5-fold cross validation for both the inner and the outer loops. We used balanced accuracy here as the primary selection criterion for the hyperparameter tuning in the inner loop. Balanced accuracy was also used for the outer loop to provide a measure of model performance. The following machine learning algorithms were used to train models: support vector machine, k-nearest neighbor, random forest, neural network, logistic regression, stochastic gradient boosting.

The best performing algorithm was then trained on the complete training data set with nested cross-validated hyperparameter tuning to model the data. To understand model performance and guard against overfitting, the model was tested against the test data set to obtain performance estimates of following metrics: weighted precision, weighted recall, balanced accuracy, Cohen κ, AUROC, and AUPRC. The final model was further assessed with a permutation test, which measured the likelihood of obtaining the observed accuracy by chance. This was done by repeating the classification (training and testing) procedure 200 times after randomly shuffling the data and permuting the labels in each iteration. The scores obtained with the permuted data were compared with the scores from the original data. We computed the probability of obtaining a score with permuted data that was better than with original data. Obtaining a small probability value rejects the null hypothesis that our model performed better than random chance and that the model had learned a real relationship between our data and dementia stages [36]. In other words, this process estimates how likely it is to obtain the observed classification performance on the test set by chance [37].

All data processing, analysis, and visualization were performed using Python (version 3.6; 64-bit) libraries (numpy, version 1.18.1; scipy, version 1.4.1; matplotlib, version 3.1.2; pandas, version 0.25.3) [38-42]. Classification algorithms were processed and analyzed using scikit-learn (version 0.22.1) and scipy [39,43]. The synthetic minority oversampling technique and neighborhood cleaning rule were implemented using imbalanced-learn (version 0.6.1) [44].

## Results

### Participants

This study used data from memory clinic (n=420), a long-term care study (n=169), and the VASPECT clinical trial (n=128) for a participant sample that allows 717 user profiles in people with clinical diagnosis and staging (Table 1) [25,26]. The mean participant age was 77.3 years (SD 10.6 years), and 59.1% of the participants were women. The mean FAST score was 4.0 (SD 0.9), and the mean GDS score was 4.8 (SD 1.9). The participants identified a median of 5 symptoms (range 1-27).

**Table 1:** Descriptive statistics of participants from clinical studies, by data source.

Characteristic		Memory clinic	Long-term care	VASPECT^a^ open-label trial	Total
Sample size, n (%)		420 (58.5)	169 (23.5)	128 (18.0)	717 (100)
Age (in years), mean (SD)		74.6 (12.5)	81.0 (19.1)	75.4 (9.2)	77.3 (10.6)
FAST, mean (SD)		4.0 (0.9)	5.3 (1.1)	4.3 (0.5)	4.1 (0.9)
GDS, mean (SD)		4.8 (1.9)	5.2 (1.0)	—^b^	5.2 (1.1)
Reported symptoms, median (range)		5 (1-14)	4 (1-12)	6 (1-27)	5 (1-27)
**Sex, n (%)**					
	Female	228 (54.3)	129 (76.3)	67 (52.3)	424 (59.1)
	Male	192 (45.7)	40 (23.7)	61 (47.7)	293 (40.9)
FAST, mean (SD)		4.0 (0.9)	5.3 (1.1)	4.3 (0.5)	4.1 (0.9)
GDS, mean (SD)		4.8 (1.9)	5.2 (1.0)	—	5.2 (1.1)
Reported symptoms, median (range)		5 (1-14)	4 (1-12)	6 (1-27)	5 (1-27)
**Reported symptoms by dementia stage, median (range)**					
	Mild cognitive impairment	3 (1-14)	2 (2-4)	—	4 (1-14)
	Mild dementia	5 (1-11)	3 (1-8)	6 (1-24)	5 (1-24)
	Moderate dementia	5.5 (1-11)	4 (1-7)	7 (2-27)	4.5 (1-27)
	Severe dementia	5 (2-11)	5 (2-12)	10 (7-13)	5 (1-13)
**Stage, n (%)**					
	Mild cognitive impairment	126 (30.0)	7 (4.1)	—	133 (18.5)
	Mild dementia	203 (48.3)	33 (19.5)	96 (75)	332 (46.3)
	Moderate dementia	58 (13.8)	50 (29.6)	30 (23.4)	138 (19.2)
	Severe dementia	33 (7.8)	79 (46.7)	2 (1.5)	114 (15.8)
**Age (years), mean (SD)**					
	Mild cognitive impairment	71.2 (14.3)	87.5 (9.9)	—	73.2 (12.2)
	Mild dementia	75.3 (11.8)	81.5 (16.3)	74.7 (8.8)	76.4 (9.8)
	Moderate dementia	77.1 (10.1)	83.6 (14.1)	77.5 (9.9)	80 (9.4)
	Severe dementia	77.3 (10.7)	78.5 (23)	75 (16.9)	81.2 (9.7)

^a^An open-label trial of donepezil in vascular and mixed dementia.

^b^No data from this source.

### Symptoms

Table 2 shows the frequency of dementia symptoms reported for user profiles and classified by dementia stage as assessed clinically with the FAST and GDS tools.

Table 2 illustrates the relationship between symptom frequency and clinical dementia stage. There was a sharp increase in the frequency of aggression, wandering, and incontinence in patients with severe dementia. By contrast, symptoms such as memory of recent events, repetitive questioning, and initiative declined with increasing dementia severity.

**Table 2:** Mean frequency of reported user profile symptoms of clinical study participants by clinically defined dementia stage.

Symptom	Mild cognitive impairment, n (%)	Mild dementia, n (%)	Moderate dementia, n (%)	Severe dementia, n (%)
Aggression	1 (0.8)	7 (2.1)	4 (2.9)	32 (28.1)
Anxiety & worry	35 (26.3)	82 (24.7)	23 (16.7)	19 (16.7)
Appetite	0 (0)	28 (8.4)	12 (8.7)	9 (7.9)
Balance	5 (3.8)	7 (2.1)	13 (9.4)	19 (16.7)
Bathing	0 (0)	9 (2.7)	11 (8.0)	6 (5.3)
Delusions & paranoia	4 (3.0)	29 (8.7)	20 (14.5)	19 (16.7)
Disorientation to place	3 (2.3)	28 (8.4)	15 (10.9)	18 (15.8)
Disorientation to time	4 (3.0)	41 (12.3)	25 (18.1)	23 (20.2)
Dressing	1 (0.8)	13 (3.9)	15 (10.9)	7 (6.1)
Eating	0 (0)	5 (1.5)	3 (2.2)	9 (7.9)
Financial management	5 (3.8)	30 (9.0)	11 (8.0)	1 (0.9)
Following instructions	6 (4.5)	12 (3.6)	2 (1.4)	2 (1.8)
Hallucinations	0 (0)	15 (4.5)	11 (8.0)	12 (10.5)
Hobbies & games	2 (1.5)	17 (5.1)	5 (3.6)	0 (0)
Household chores	4 (3.0)	39 (11.7)	13 (9.4)	5 (4.4)
Incontinence	1 (0.8)	6 (1.8)	6 (4.3)	23 (20.2)
Insight	4 (3.0)	19 (5.7)	6 (4.3)	2 (1.8)
Interest initiative	46 (34.6)	148 (44.6)	39 (28.3)	19 (16.7)
Irritability frustration	19 (14.3)	92 (27.7)	24 (17.4)	28 (24.6)
Judgment	13 (9.8)	42 (12.7)	24 (17.4)	25 (21.9)
Language difficulty	18 (13.5)	59 (17.8)	15 (10.9)	21 (18.4)
Low mood	3 (2.3)	44 (13.3)	13 (9.4)	6 (5.3)
Meal preparation cooking	10 (7.5)	57 (17.2)	19 (13.8)	2 (1.8)
Memory for names faces	2 (1.5)	29 (8.7)	24 (17.4)	12 (10.5)
Memory of future events	0 (0)	37 (11.1)	16 (11.6)	1 (0.9)
Memory of past events	8 (6.0)	33 (9.9)	21 (15.2)	13 (11.4)
Memory of recent events	100 (75.2)	233 (70.2)	81 (58.7)	30 (26.3)
Misplacing or losing objects	21 (15.8)	53 (16.0)	11 (8)	5 (4.4)
Mobility	4 (3.0)	14 (4.2)	16 (11.6)	30 (26.3)
Operating gadgets/appliances	8 (6.0)	76 (22.9)	24 (17.4)	4 (3.5)
Personal care hygiene	7 (5.3)	26 (7.8)	36 (26.1)	28 (24.6)
Physical complaints	2 (1.5)	15 (4.5)	7 (5.1)	4 (3.5)
Repetitive questions stories	51 (38.3)	169 (50.9)	50 (36.2)	18 (15.8)
Shadowing	1 (0.8)	8 (2.4)	8 (5.8)	4 (3.5)
Social interaction/withdrawal	20 (15.0)	64 (19.3)	22 (15.9)	11 (9.6)
Wandering	0 (0)	2 (0.6)	10 (7.2)	29 (25.4)

### Model Selection

Six machine learning models were tested on the training data set. Table 3 illustrates the models used and the validation data obtained for each model in terms of accuracy, precision, and recall when predicting dementia stage. The table also indicates values for the Cohen κ, which measures the agreement between the dementia stage predicted by the model and the dementia stage as determined clinically. At the end of the model selection process, the model trained with a support vector machine was selected as the best performing model when used with the training data set (Table 3).

**Table 3:** Performance of candidate models with the training data set.

Model	Balanced accuracy, mean (SD)	Precision (weighted), mean (SD)	Recall (weighted), mean (SD)	Cohen κ, mean (SD)
Support vector machine	0.73 (0.07)	0.75 (0.07)	0.75 (0.06)	0.65 (0.09)
k-nearest neighbor	0.72 (0.08)	0.73 (0.07)	0.72 (0.07)	0.62 (0.10)
Random forest	0.70 (0.07)	0.74 (0.08)	0.73 (0.07)	0.62 (0.09)
Neural network	0.66 (0.10)	0.67 (0.11)	0.66 (0.09)	0.54 (0.13)
Logistic regression	0.65 (0.08)	0.66 (0.08)	0.66 (0.08)	0.53 (0.10)
Gradient boosting	0.68 (0.07)	0.70 (0.07)	0.70 (0.07)	0.58 (0.10)

Next, the support vector machine was trained and optimized with a nested cross-validated grid search on the complete training set. The final trained model was used with the test data set to obtain performance metrics for this new data subset (balanced accuracy 0.85; AUROC 0.96, weighted precision 0.87; weighted recall 0.86; AUPRC 0.91), indicating excellent model performance.

### Final Model Prediction Based on Dementia Stage

The ability of the support vector machine model to predict each of the 4 dementia stages showed excellent precision and recall for all dementia stages (Table 4).

**Figure 2:**
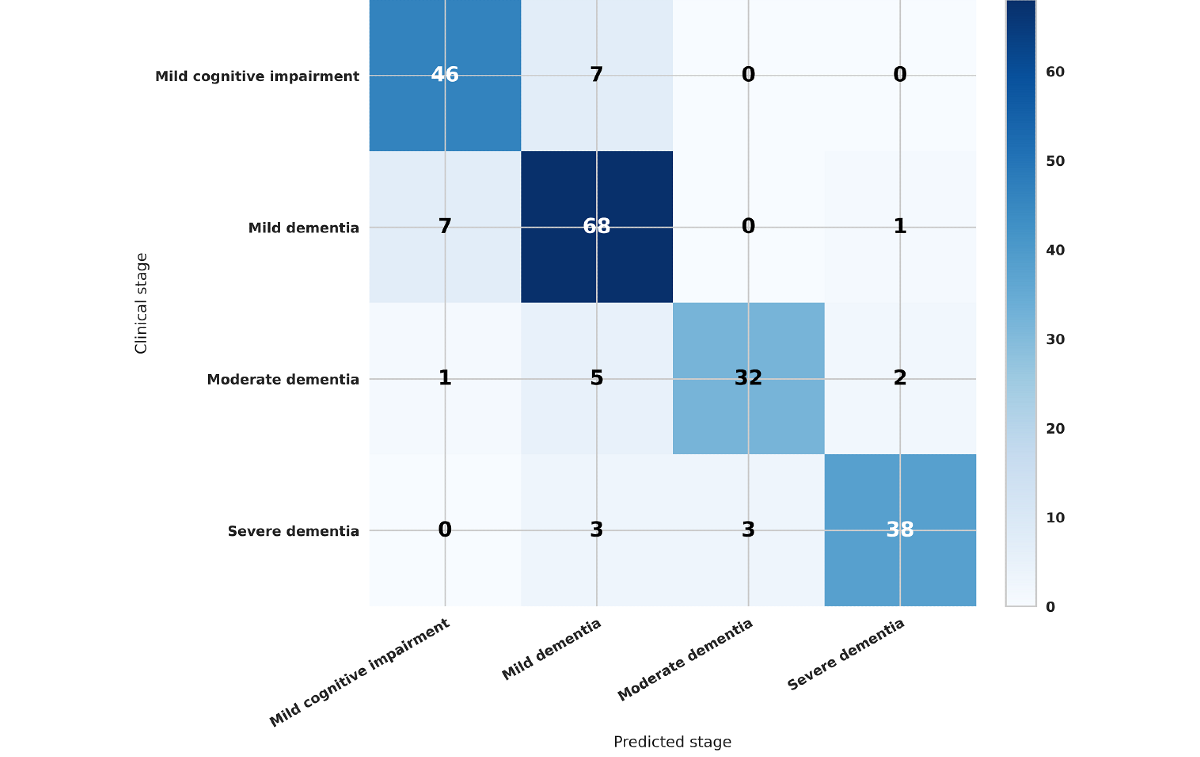
Confusion matrix of the trained model. Each row of the matrix represents the instances of actual dementia stage while each column represents the instances of predicted dementia stage. Counts are colored from the highest cell (darker) to the lowest (lighter). The top-left to bottom-right diagonal cells count correctly predicted dementia stages.

To better demonstrate predictions made across the dementia stages by the model, a confusion matrix is presented in Figure 2.

**Figure 3:**
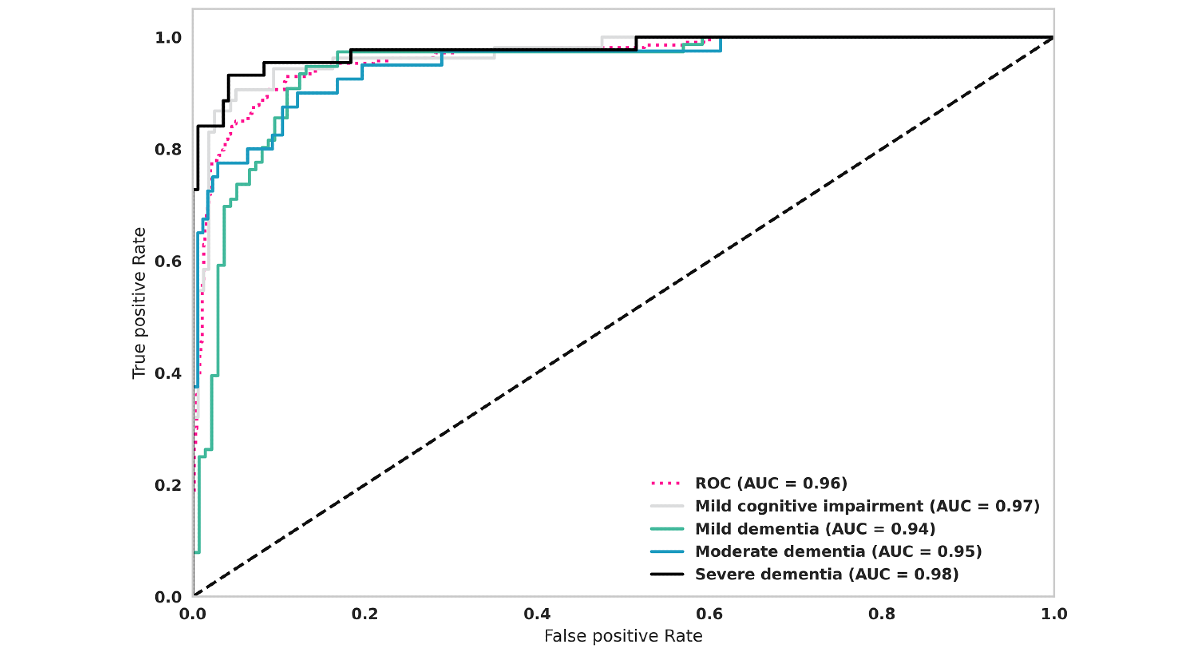
Receiver operating characteristic curves for each dementia stage predicted by the model. AUC: area under the curve; ROC: receiver operating characteristics.

To determine the relationship between the true positives and false positives identified by the model, receiver operating characteristic curves of the model’s output were plotted (Figure 3). The AUROC for the overall model was high (AUROC 0.96). The final model achieved the best results when classifying severe dementia (AUROC 0.98) and mild cognitive impairment (AUROC 0.97).

**Table 4:** Precision and recall of model prediction by dementia stage.

Stage	Precision	Recall
Mild cognitive impairment	0.85	0.87
Mild dementia	0.82	0.89
Moderate dementia	0.91	0.80
Severe dementia	0.93	0.86

**Figure 4:**
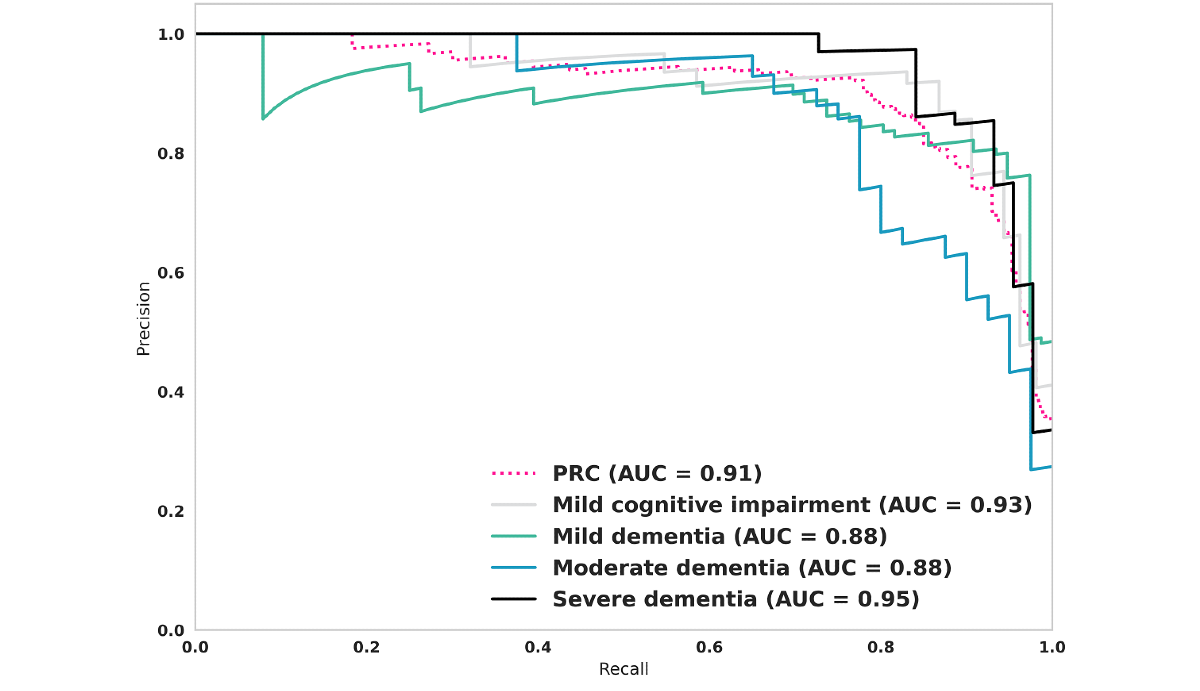
Precision-recall curves for each dementia stage as predicted by the model. AUC: area under the curve; PRC: precision-recall curve.

Another way to assess the relationship between false positives and false negatives is to use a precision-recall curve, where high precision indicates a low false positive rate, and high recall denotes a low false negative rate. Figure 4 shows precision-recall curves of the overall model output by dementia stage. The overall model performed well (AUPRC 0.91). When AUPRC metrics were compared for individual dementia stages, the model performed best when classifying severe dementia (AUPRC 0.95) and mild cognitive impairment (AUPRC 0.93). It was somewhat less able to discriminate between mild and moderate dementia (Figure 4). These observations are similar to those seen when these relationships were evaluated with receiver operating characteristic curves as shown in Figure 3.

**Figure 5:**
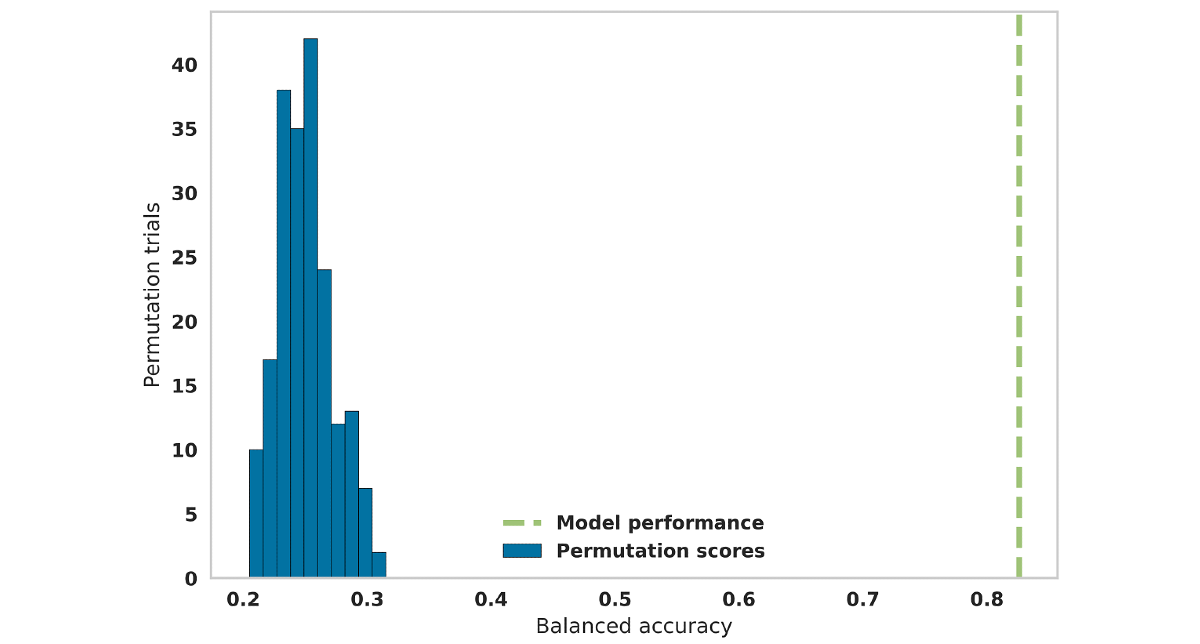
The classification scores obtained from models trained on permuted data were well short of scores obtained with the model trained on original data.

To confirm that the model could accurately predict dementia stage, we performed a permutation test, where we used randomly mislabeled data in several iterations, grouped about the level expected by chance (Figure 5). The random permutation scores had a balanced accuracy between 0.2 and 0.3. This was well short of the classification score for the actual data, which had a balanced accuracy of 0.85, and a probability of obtaining this by chance <.005.

## Discussion

This study aimed to stage dementia severity based on symptom profiles constructed with a standardized symptom menu from an online symptom tracking tool. We found that a support vector machine model consistently predicted each of the 4 dementia stages based on online symptom data reported by caregivers of persons with dementia. This approach to staging dementia severity will allow us to gain insights from online reported symptom data that can be collected by SymptomGuide Dementia and other similar platforms. In this way, symptom reporting can facilitate understanding dementia progression. For example, earlier work from this database suggests important qualitative differences in symptoms such as misplacing objects (eg, with dementia progression, less instances of simply forgetting where an item might be and more instances of placing items in an odd place [6]) or verbal repetition (eg, repetitive questioning, most often seen in mild dementia; it is characteristically dementia-defining when seen with early functional decline—difficulty operating familiar gadgets or appliances [5]). In this way, allowing the patients and carer voices to contribute to our understanding of dementia phenomenology can lead to recognizing patterns of both progression—as above—and of treatment [13]. The updated staging algorithm described here will further such inquiries.

We trained multiple machine learning algorithms and selected the best performing algorithm to use for our dementia stage classification task. A support vector machine model using a one-versus-rest approach demonstrated the best performance during model selection trials. The selected algorithm was then trained on the complete training data and validated using a test data set. The final model demonstrated excellent performance in discriminating dementia stages (balanced accuracy 0.85, AUROC 0.96). Receiver operating characteristic curves tend to present an optimistic picture of performance when the data set has a skewed distribution of the target variable [45]. For this reason, the performance of the model was also assessed with precision-recall curves. These too demonstrated that the model performed well, especially when classifying severe dementia. Since mild cognitive impairment and severe dementia can be considered bookends to the dementia spectrum, we can be reassured of both the model’s precision and recall in classifying these extremes. For example, our model correctly classified a 75-year-old participant who reported 4 symptoms (social interaction/withdrawal, irritability and frustration, interest and initiative, aggression) as having mild cognitive impairment and a 76-year-old participant as having severe dementia based on a different set of 3 symptoms (wandering, delusions and paranoia, and aggression). The model was somewhat less accurate when classifying mild and moderate dementia. This is perhaps not surprising as symptom profiles in the middle of the dementia spectrum can exhibit a higher degree of overlap and can be difficult to distinguish clinically as well [46].

The very low probability value from the permutation tests (<.005) reassures us that the model learned a real relationship between the data and dementia stages. It demonstrates that the classification performance of the model with respect to the test set is unlikely to have occurred as a result of chance.

Our data must be interpreted with caution. For model stability, symptoms were eliminated based on a set threshold of occurrence. While this worked well here, it might not hold in a larger data set. In addition, we used 3 separate data sets that used variations of our standardized symptom menu, with differences in the composition and order of presentation of the symptoms. Since most of these patient symptom profiles were constructed with the supervision of a clinician or a rater, the model may be less generalizable to web-based symptom profiles constructed without clinician facilitation or guidance.

Several other recent studies have applied machine learning algorithms for dementia research [47-51]. Most have used neuroimaging or biomarker data to train these models. Most models trained with neuroimaging data focus on distinguishing individual patients from healthy controls, whereas our model distinguished between different stages of dementia severity [52]. Extraction of image characteristics from neuroimaging data can be susceptible to variations in the scanner hardware and image acquisition protocols. This can produce models that may not be generalizable when applied to data acquired from different imaging sources [52]. Additionally, scans such as amyloid positron emission tomography imaging, used for diagnostic certainty regarding Alzheimer disease, can cost upward of US $4000. Machine learning models that do not rely neuroimaging data to stage or diagnose dementia, if used clinically, can potentially reduce the number of participants that require expensive neuroimaging tests [53].

More recent studies have also used data extracted from electronic health records which may include structured and unstructured data such as clinical notes, drug prescriptions, and diagnosis codes to develop predictive models [54-60]. These models have been trained to predict future onset of dementia [53-56] or diagnose undetected dementia [57,58,60] with varying levels of accuracy and can potentially serve as case-finding algorithms to target high-risk patients with further clinical assessments to confirm dementia diagnosis [58]. However, these models are contingent on the availability of consolidated electronic health records, sufficient health care interactions by the patient, and correctly transcribed notes and diagnosis codes [55,57,61]. In contrast, the model developed here does not use data extracted from electronic health records, rather it predicts dementia severity based on self-reported caregiver data and can be used to potentially unlock insights from online self-reported symptoms.

Few studies have used machine learning models to stage the severity of dementia or differentiate types of dementia [62]. One such study uses a combination of cognitive function tests and clinicians’ assessments of patients to assess dementia severity on the Clinical Dementia Rating Scale [63]. On the other hand, a combination of neuropsychiatric assessment, mental status examination, and laboratory investigations have also been used to classify dementia severity with a high degree of accuracy [64]. Such approaches require trained interviewers and clinician assessment to obtain input data for the predictive models. This is in contrast to the model developed here, which is designed to stage dementia severity based on self-reported data thereby potentially offering a more economically viable screening tool for dementia severity.

Even though our sample size (n=717) is relatively small, it is larger than that of other machine learning studies in dementia, except for a 2019 report that used administrative data to diagnose incident dementia [47]. The advantage of utilizing patient reported outcomes such as SymptomGuide is that it reflects the lived experience of the patient or caregiver and focuses on what is meaningful to them. It is easier to source and computationally less expensive to train models when compared to imaging data or complex biomarkers [48,49]. Interestingly, Chiu et al [50] reported that a machine learning algorithm could be used to derive a screening instrument to distinguish normal cognition, mild cognitive impairment, and dementia. This further emphasizes that dementia symptoms can be used with machine learning to characterize various stages of dementia. On the other hand, our approach used a patient-derived library of symptoms to train a machine learning model, whereas Chui et al used machine learning to reduce the dimensionality of their screening instrument [50]. It is likely that, given the high dimensionality of late-life dementia, different machine learning approaches may be useful in dementia research. In our earlier work, we developed a model based on a neural network trained on 320 symptom profiles reported by caregivers of persons with dementia [24]. This study expands on our previous work by increasing the sample size and diversity of the training data. We also examined the performance of multiple machine learning algorithms on the available data to maximize our interpretation. The support vector machine outperformed the neural network approach, highlighting the advantage of the current approach.

Future studies could include integrating the model developed here with an electronic interface by which end users could build a symptom profile and obtain the dementia stage. This instrument also has the potential to facilitate physician-patient discussions or to aid screening patients before their in-person memory clinic visit. This model can potentially be applied on other web-based data sets that contain symptom profiles of persons affected with dementia.

The model presented here can classify dementia stages from individualized symptom data. This real-world evidence will enable us to better understand the symptoms that matter most to people affected by dementia at each dementia stage. That information can greatly expand access to understanding the lived experience of dementia.

## Abbreviations

AUROC: area under the receiver operating characteristic curve
AUPRC: area under the precision-recall curve
FAST: Functional Assessment Staging Test
GDS: Global Deterioration Scale
